# The Mechanism of Antifungal Action of a New Polyene Macrolide Antibiotic Antifungalmycin 702 from *Streptomyces* *padanus* JAU4234 on the Rice Sheath Blight Pathogen *Rhizoctonia solani*

**DOI:** 10.1371/journal.pone.0073884

**Published:** 2013-08-12

**Authors:** Zhi-Qiang Xiong, Xiao-Rong Tu, Sai-Jin Wei, Lin Huang, Xun-Hang Li, Hui Lu, Guo-Quan Tu

**Affiliations:** 1 College of Bioscience and Bioengineering, Jiangxi Agricultural University, Nanchang, China; 2 Key Laboratory of Synthetic Biology, Institute of Plant Physiology and Ecology, Shanghai Institutes for Biological Sciences, Chinese Academy of Sciences, Shanghai, China; University Paris South, France

## Abstract

Antifungalmycin 702, a new polyene macrolide antibiotic produced by *Streptomyces* *padanus* JAU4234, has a broad antifungal activity and may have potential future agricultural and/or clinical applications. However, the mechanism of antifungal action of antifungalmycin 702 remains unknown. Antifungalmycin 702 strongly inhibited mycelial growth and sclerotia formation/germination of *Rhizoctonia solani*. When treated with antifungalmycin 702, the hyphae morphology of *R* *. solani* became more irregular. The membrane and the cellular organelles were disrupted and there were many vacuoles in the cellular space. The lesion in the plasma membrane was detected through the increase of membrane permeability, lipid peroxidation and leakage of cell constituents. In summary, antifungalmycin 702 may exert its antifungal activity against *R* *. solani* by changing the structure of cell membranes and the cytoskeleton and interacting with the organelles.

## Introduction

The basidiomycete fungus *Rhizoctonia solani* is an economically important pathogen with a broad host range and worldwide distribution which causes the most destructive rice sheath blight and severely lowers both rice yield and quality in China and other rice-growing countries. Only in Eastern Asia, it affects 15–20 million ha of paddy-irrigated rice and causes a yield loss of 6 million tons of rice grains per year [1]. *R* *. solani* is also considered to be the most destructive pathogen for other economic crops such as soybean (*Glycine max*) and corn (*Zea mays*) [1]. Biocontrol is a better method against *R* *. solani* , while its effectiveness is often strongly affected by environmental conditions [2]. Using chemical fungicide is the most common strategy to effectively minimize the severity of *R* *. solani* , but is not considered to be a long term solution because of the potential health and environmental risks [3]. Moreover, pathogenic microorganisms are rapidly adapting to fungicides, making them ineffective and leading to the emergence of resistance. Hence, one direct course of action to control fungicide-resistant pathogenic infections and to reduce the negative environmental impact because of the use of chemicals is to discover new fungicides. Therefore, searching for novel natural product-based fungicide as a sustainable biotechnological alternative has gained momentum in the past decades.

**Figure 1:**
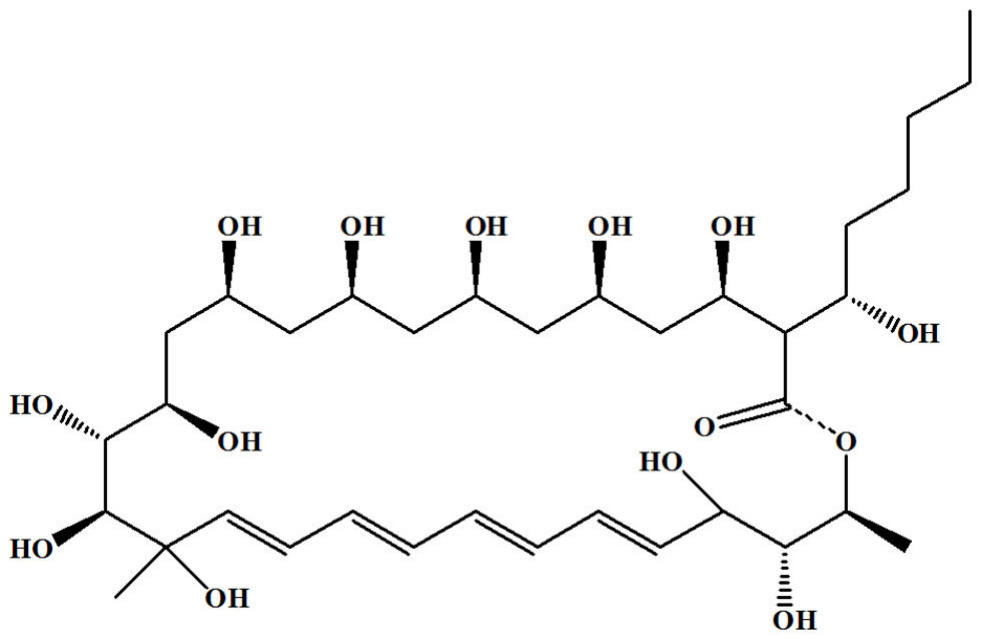
Chemical structure of antifungalmycin 702.

Polyene macrolide antibiotics, one of the most important subgroup of polyketides, possess a typical polyene structure ranging from three to seven double bonds in length. To date, more than 200 known polyene macrolide antibiotics (e.g., rapamycin, nystatins, filipins and amphotericin B) have been isolated and characterized, most of which are produced by the genus *Streptomyces* [4]. In the course of our screening program for new microbial natural products, we isolated a new polyene macrolide antibiotic antifungalmycin 702 from *S* *. padanus* JAU4234. Antifungalmycin 702, possessing a macrocyclic lactone ring with four double bonds (Figure 1), has good antifungal activity and may have potential future agricultural/clinical application e.g., as a fungicide [5]. Here, we first reported the mechanism of antifungal action of antifungalmycin 702 on *R* *. solani* AG1 IA.

## Materials and Methods

### Strain, antimicrobial agent and reagents

*R* *. solani* AG1 IA was maintained on PDA (potato dextrose agar) medium by our laboratory. Antifungalmycin 702 was purified according to the method described by Xiong et al. [5]. Antifungalmycin 702 solutions used for biological assay were prepared by diluting the stock solution (100 mg/ml) in water to the required concentrations. Validamycin, tannic acid, congo red, crystal violet, glutaraldehyde, osmium tetroxide, PBS buffer, and other chemicals were purchased from Sigma-Aldrich (St. Louis, MO, USA).

### Morphology of *R* *. solani*

After treated with the negative control PBS buffer (0.1 M, pH 7.0) or 3.35 µg/ml antifungalmycin 702, the samples of *R* *. solani* were visualized by optical microscopy (OM), scanning electron microscopy (SEM) and transmission electron microscopy (TEM). (a) OM: *R* *. solani* mycelia were stained by cell wall staining solution (10% tannic acid, 5% Congo red, and 5% crystal violet) at room temperature for 5 min, washed with water, and examined by a Zeiss OM with a MC 80 camera. (b) SEM: *R* *. solani* cells were incubated with the control PBS buffer or 3.35 µg/ml antifungalmycin 702 at 28° C for 2 h and fixed with an equal volume of 5% (w/v) glutaraldehyde for 2 h at 4° C, and then washed with 0.1 M cacodylate buffer, pH 7.4. The prepared samples were treated with 1% (w/v) osmium tetroxide, washed with 5% (w/v) sucrose in cacodylate buffer, and subsequently dehydrated in a graded ethanol series (30%, 70%, 80%, 90%, 100%, and 100% EtOH). After lyophilization using an Hitachi ZS-2030 critical point dryer and gold coating using a Hitachi Z-1010 Sputtering System, the samples were visualized by Hitachi S-3000N SEM [5]. (c) TEM: samples were fixed with 2.5% glutaraldehyde, post-fixed with 1% osmium tetroxide followed by 1% uranyl acetate, dehydrated through a graded series of ethanol, and embedded in EPON-812 resin. Ultra-thin sections were stained with uranyl acetate followed by lead citrate, and viewed on an Hitachi H-7650 TEM [3].

### Suppression of mycelial growth of *R* *. solani* by antifungalmycin 702

Effective concentrations of antifungalmycin 702 that result in 50% and 90% inhibition (EC_50_ and EC_90_) on *R* *. solani* were bioassayed on PDA in Petri dishes with validamycin as a positive control [6]. Antifungalmycin 702 were mixed with PDA to produce a series of concentrations (0-13.71 µg/ml) in the final test solution. The 5-mm-diameter inoculum plugs of *R* *. solani* removed from the margin of a 4-day-old colony on PDA were placed at the center of the dishes. Linear growth of *R* *. solani* at 28° C was recorded 2 days after treatment. Each treatment consisted of three replicates. Inhibition percentage was obtained from the equation: Inhibition (%) = [(growth diameter in untreated control- growth diameter intreatment) ×100]/ growth diameter in untreated control [6]. The experiment was repeated twice.

### Effect of antifungalmycin 702 on the formation of sclerotia

To test antifungalmycin 702 effect on the formation of sclerotia of *R* *. solani* , antifungalmycin 702 were mixed with PDA to produce a series of different final concentrations (0-48 µg/ml). A 5-mm inoculum plug was inoculated with the mycelium at the center of the dishes. The number of sclerotia was counted and the average number of the three PDA plates was calculated after 3-13 days at 28° C [7]. There were nine dishes (replicates) for each treatment. The experiments were repeated for three times.

### Suppression of myceliogenic germination of sclerotia of *R* *. solani* by antifungalmycin 702

Mycelial plugs of *R* *. solani* were inoculated on PDA in petri dishes and incubated at 28° C. After 14 days, sclerotia of *R* *. solani* formed in each dish were harvested and air-dried at room temperature. Sixty sclerotia of *R* *. solani* were submerged in 20 ml antifungalmycin 702 solutions (0-48 µg/ml) for 24 h (submerged in distilled water as a control). Then the sclerotia were rinsed in sterile distilled water for three times (1 min each time) and individually inoculated on PDA in petri dishes (four sclerotia per dish). The sclerotial germination and the colony diameter of *R* *. solani* around each sclerotium were recorded after incubation at 28° C for 2, 3 and 4 days. A sclerotium was considered to have germinated when white and cottony mycelia appeared on the sclerotial surface or on the agar medium [7]. The experiment was repeated for three times.

### Effect of antifungalmycin 702 on the physicochemical and physiological changes of *R* *. solani*

The hyphae were incubated in PDB for 2 days at 28° C and centrifuged at 12 000 r/min for 10 min at 4° C. The hyphae were rinsed with deionized water and centrifuged under the same conditions as above for two times. Then the biomass was suspended in the deionized water or in the antifungalmycin 702 solutions and incubated in the rotating shaker at 160 r/min and 28° C for different time. Intracellular chitnase activity and N-acetylglucosamine content were assayed in accordance with the methods of Hoster et al. [8] and Escalante et al. [9], respectively. The membrane permeability was represented by the change of electrical conductivity of broth [9] using a conductimeter (DDS-307A, Shanghai Precision and Scientific Instrument, Shanghai, China). The leakage of protein and sugar were analyzed according to the methods described by Liu et al. [10]. Ergosterol content in the plasma membrane was detected according to the method described by Tian et al. [11]. Malondialdehyde was determined by 2-thiobarbituric acid method [12]. The data were obtained by repeating for three times.

### Statistical analysis

Each experiment was performed at least three technical replicates. The error bar indicates standard deviation (SD). The statistically significant difference (P <0.05) between the control and the tested groups was analyzed by SPSS software (version 11.5, SPSS Inc, USA).

## Results and Discussion

**Figure 2:**
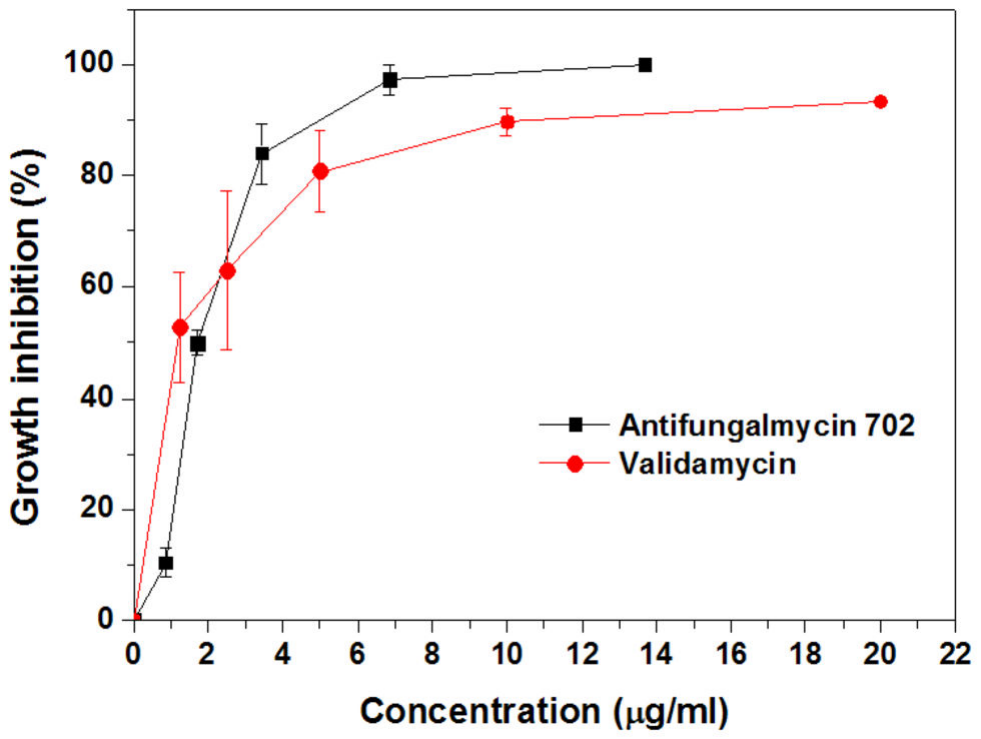
Effect of antifungalmycin 702 and validamycin (the positive control) on inhibition of mycelial growth of *R* *. solani* .

The disease cycle of *R* *. solani* is important in regards to management and control of the pathogen. *R* *. solani* exists primarily as vegetative mycelium and/or sclerotia [1]. Antifungalmycin 702 significantly inhibited the mycelial growth of *R* *. solani* and showed dose-dependent inhibitory effect with EC_50_ and EC_90_ values of 1.81 µg/ml and 3.35 µg/ml, respectively (Figure 2). Validamycin, a known antibiotic and fungicide, is widely used as a crop protectant for the control of *R* *. solani* in many Asian countries [13]. As shown in Figure 2, antifungalmycin has a good inhibition efficiency compared to validamycin (EC_50_ and EC_90_ values of 1.26 µg/ml and 4.57 µg/ml against *R* *. solani* ). Moreover, antifungalmycin 702 displayed not only the significant inhibition to the number of sclerotia but the increase of formation time of sclerotia (Table 1). Significant reduction of the formation rate of sclerotia was observed at concentrations of above 3 µg/ml for antifungalmycin 702. Germination rate of sclerotia was also suppressed by antifungalmycin 702 (Table 2). When treated with >41.13 µg/ml of antifungalmycin 702, myceliogenic germination of sclerotia was not viable after 72 h incubation.

**Table 1:** Effect of antifungalmycin 702 on the formation of sclerotia of *Rhizoctonia solani*.

**Concentration (μg/ml)**	**Time of Sclerotia formation (h)**	**Number of sclerotia**	**Rate of Sclerotia formation (%)**
**CK**	96	102.0±7.21	/
**0.75**	96	97.0±2.65	95.10
**1.50**	96	84.7±8.02	83.04
**3.00**	120	61.7±4.73	60.49
**6.00**	144	59.3±5.51	58.14
**12.00**	192	52.7±4.04	51.67
**24.00**	240	48.0±3.61	47.06
**48.00**	312	40.0±4.36	39.21

CK: the control, 0 µg/ml antifungalmycin 702.

**Table 2:** Effect of antifungalmycin 702 on myceliogenic germination of sclerotia.

Antifungalmycin 702 (μg/ml)	Number of myceliogenically germinated sclerotia (n=60) after incubation for					
	24h		48h		72h	
	Germination number	Germination rate (%)	Germination number	Germination rate (%)	Germination number	Germination rate (%)
CK	60	100	60	100	60	100
5.14	59	98.33	60	100	60	100
10.28	58	96.67	60	100	60	100
20.57	52	86.67	57	95	58	96.67
33.51	0	0	0	0	4	6.67
41.13	0	0	0	0	0	0
48.00	0	0	0	0	0	0

CK: the control, 0 µg/ml antifungalmycin 702.

**Figure 3:**
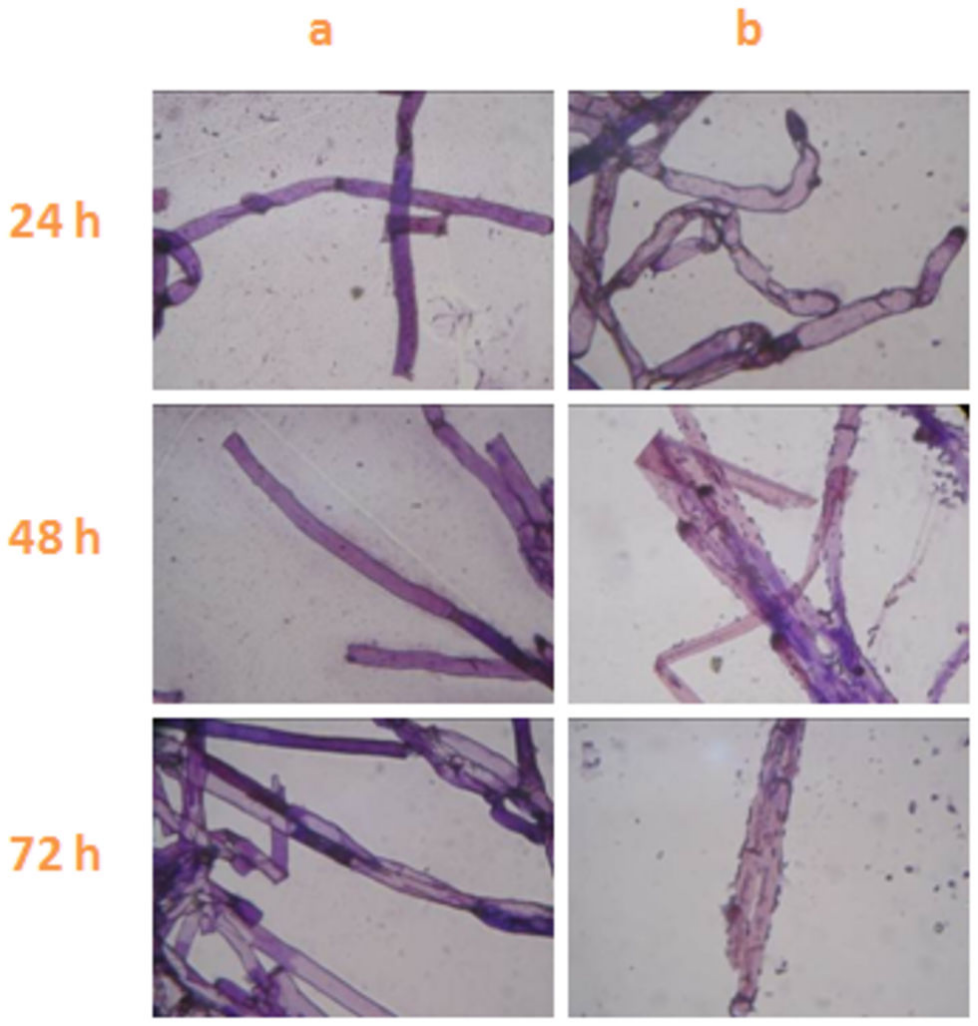
Morphology of hyphae treated with PBS buffer (a) and 3.35 µg/ml antifungalmycin 702 (b) at 28° C for 24 h, 48h, and 72h.

**Figure 4:**
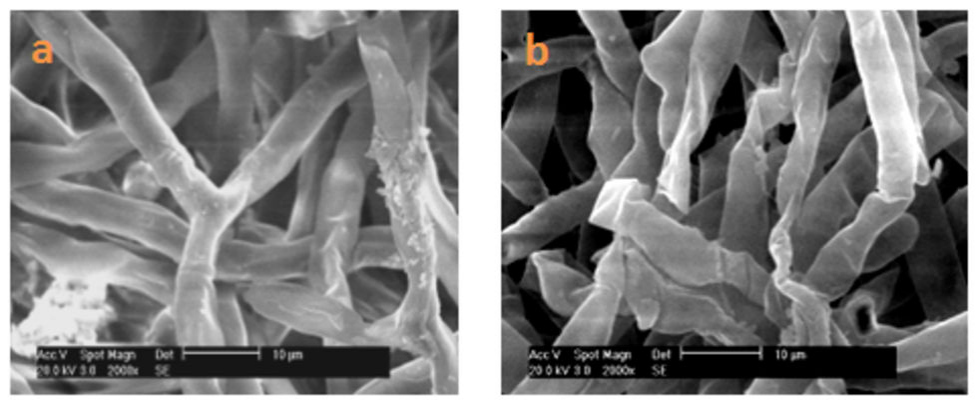
Scanning electron microscopy (2, 000 X) of *R* *. solani* treated with PBS buffer (a) and 3.35 µg/ml antifungalmycin 702 (b). Bar: 10 µm.

**Figure 5:**
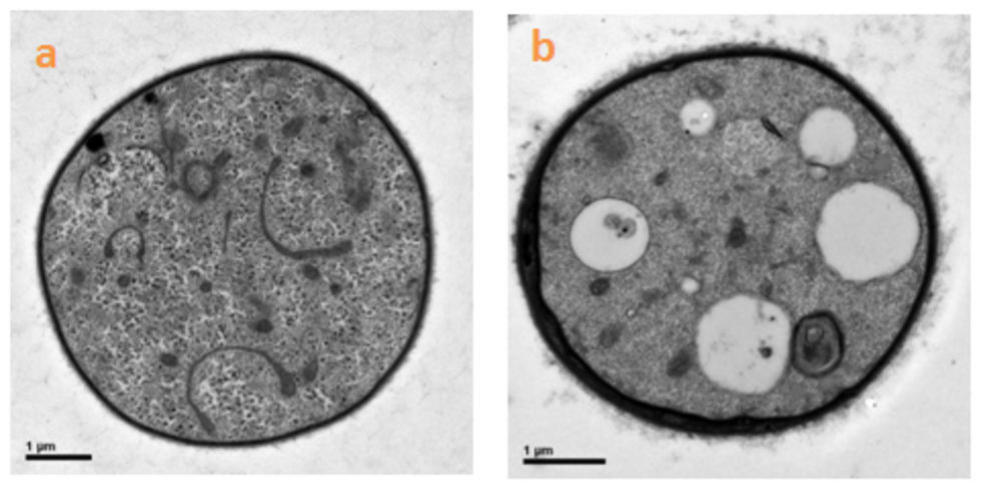
Transmission electron micrographs (20, 000 X) of *R* *. solani* cellular organelles treated with PBS buffer (a) and 3.35 µg/ml antifungalmycin 702 (b). Bar: 1 µm.

Based on OM and SEM assessments of the hyphal morphology, the control mycelia had regular hyphae with a smooth surface (Figures 3a and 4a), but antifungalmycin 702 caused the increased hyphae branching and thinner appearance of hyphae with a wrinkled surface (Figures 3b and 4b). In TEM photographs, the cell wall, cell membrane and cellular organelles were clearly seen in untreated hyphae (Figure 5a), while cell wall of hyphae treated with antifungalmycin 702 may be degraded and the cell membrane became unclear (Figure 5b). The space between the cell wall and cell membrane widened. The cell organelles were also seriously destroyed. There were inner vacuoles in treated cells while they were not seen in untreated cells. These results suggest that antifungalmycin 702 may disrupt the cell membrane and then interact with cellular organelles.

**Figure 6:**
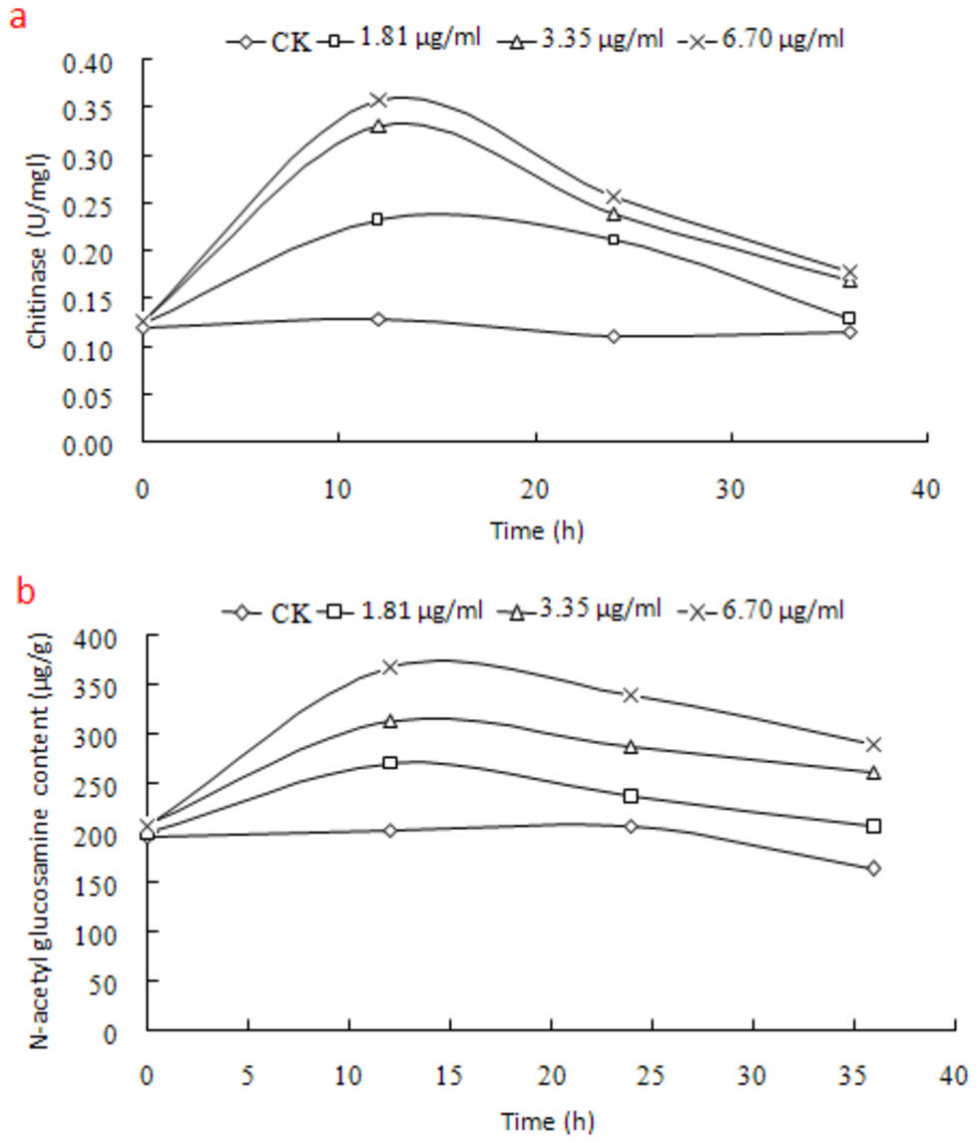
Effect of antifungalmycin 702 on chitinase (a) and N-acetyl glucosamine content (b) of *R* *. solani* .

**Figure 7:**
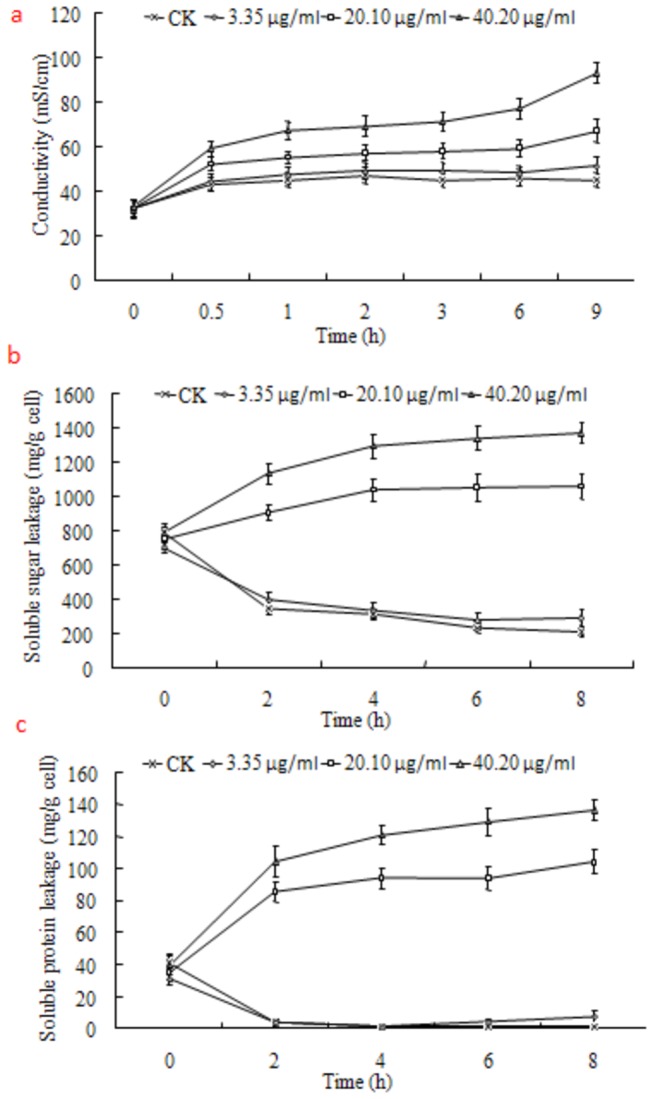
Effect of antifungalmycin 702 on membrane permeability (a), soluble sugar leakage (b), and soluble protein leakage (c) of *R* *. solani* .

**Figure 8:**
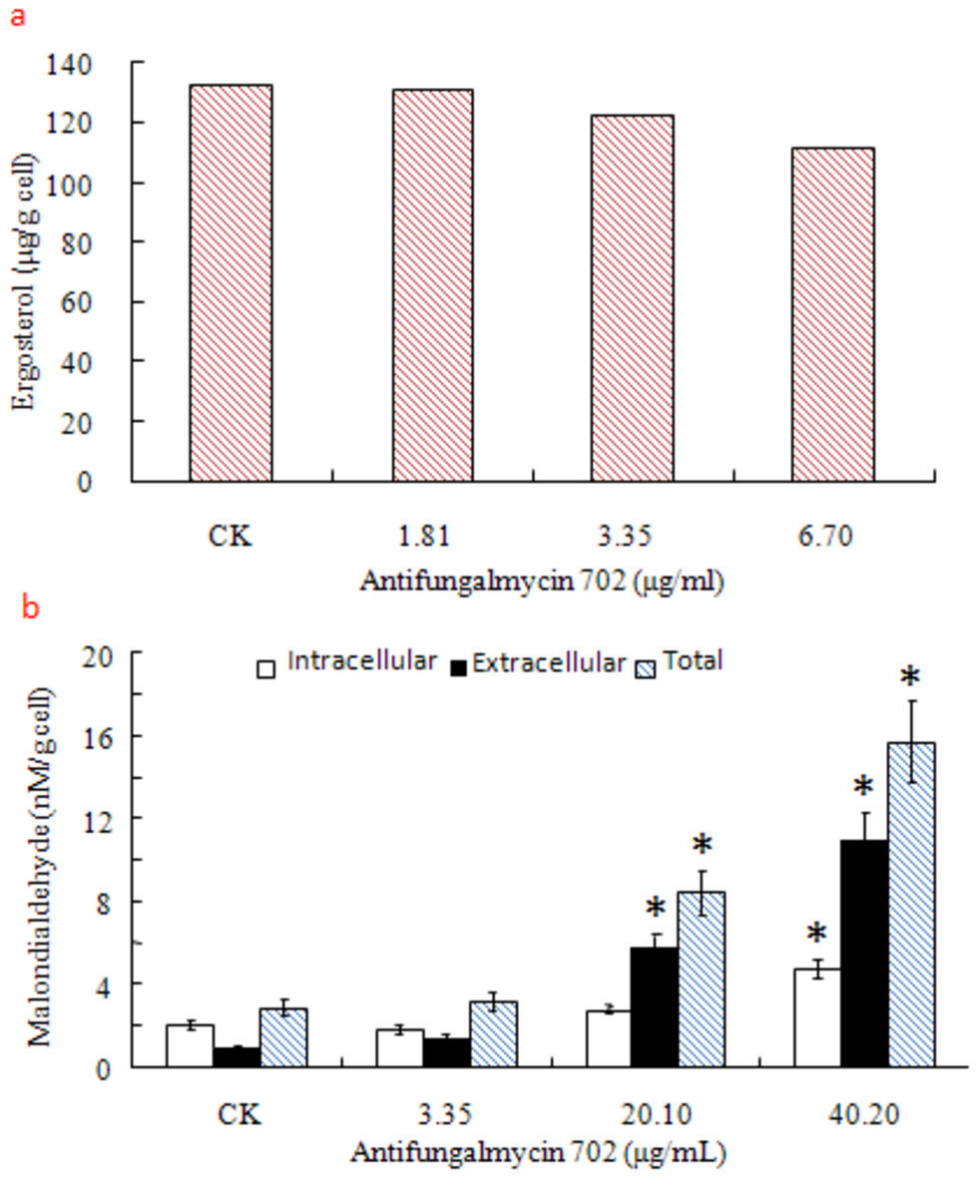
Effect of antifungalmycin 702 on ergosterol (a) and malondialdehyde (b) content of *R* *. solani* . Asterisk denotes a significant difference (P <0.05) compared with CK (the control, 0 µg/ml antifungalmycin 702).

As chitin is the main component of the cell walls of fungi, chitinase is generally found in fungi to decompose chitin to N-acetylglucosamine [8]. Compared with the untreated cells, chitnase activity and N-acetylglucosamine content were significantly enhanced with the increase of antifungalmycin 702 concentration in treated cells (Figure 6), suggesting that antifungalmycin 702 may induce hydrolytic enzymes’ activities to degrade cell wall. In addition, the membrane permeability of *R* *. solani* was investigated with the change of electrical conductivity of broth. The treated cells had higher conductivity compared to the control (Figure 7a). The conductivity increased with the time of incubation and the increase of antifungalmycin 702 concentration, indicating that the cell membrane is disrupted by antifungalmycin 702. This hypothesis was also demonstrated by the significant increase of soluble sugar and protein leakage (Figure 7b and 7c). Ergosterol is the major sterol component of the fungal cell membranes responsible for maintaining cell function and integrity [11]. After the incubation of *R* *. solani* at 6.7 µg/ml antifungalmycin 702, a reduction (15.4%) of ergosterol content was observed in the plasma membrane, suggesting that antifungalmycin 702 may interact with ergosterol which leads to depletion of total ergosterol in the cells (Figure 8a). Malondialdehyde is an indicator of lipid peroxidation in cell, and its level directly reflects the extent of damage in lipid membranes [12]. Intracellular and extracellular malondialdehyde content were gradually increased along with the successive increase of antifungalmycin 702 concentration (Figure 8b), indicating that antifungalmycin 702 induces lipid peroxidative damage to cell membrane and results in the malondialdehyde leakage from cells.

The polyene antibiotics’ mechanism of action is commonly a specific interaction with membrane sterols that results in a changed permeability [14,15]. Our results suggest that, like other polyene macrolide antibiotics [15,16], antifungalmycin 702 may exert its antifungal activity by disrupting the structure of cell membranes and the cytoskeleton and interacts with the organelles. Antifungalmycin 702 does not induce acute lethal toxicity at 1,500 mg/kg by the intraperitoneal and oral routes of administration in mice, suggesting that it may have a low toxicity [5]. In summary, owing to the mechanism of action and toxicological/antifungal characteristics [5] of antifungalmycin 702, it has great potential for agricultural/clinical application in the treatment of fungal pathogen infections.
